# Effects of a Multispecies Probiotic Mixture on Glycemic Control and Inflammatory Status in Women with Gestational Diabetes: A Randomized Controlled Clinical Trial

**DOI:** 10.1155/2016/5190846

**Published:** 2016-06-26

**Authors:** Sadegh Jafarnejad, Sadaf Saremi, Farzan Jafarnejad, Arman Arab

**Affiliations:** ^1^Department of Clinical Nutrition, School of Nutritional Sciences and Dietetic, Tehran University of Medical Sciences, P.O. Box 14155-6117, Tehran, Iran; ^2^Department of Public Health, Isfahan University of Medical Sciences, Isfahan, Iran; ^3^Department of Health Information Management, Mazandaran University of Medical Sciences, Sari, Iran; ^4^Department of Community Nutrition, School of Nutrition and Food Science, Isfahan University of Medical Sciences, Isfahan, Iran

## Abstract

*Objective*. This trial aims to examine the effects of a Probiotic Mixture (VSL#3) on glycemic status and inflammatory markers, in women with GDM.* Materials and Methods.* Over a period of 8 weeks, 82 women with gestational diabetes were randomly assigned to either an intervention group (*n* = 41) which were given VSL#3 capsule or to a control group which were given placebo capsule (*n* = 41). Fasting plasma glucose, homeostatic model assessment of insulin resistance, glycosylated hemoglobin, high-sensitivity C-reactive protein, tumor necrosis factor-*α*, interleukin-6, Interferon gamma, and interleukin-10 were measured before and after the intervention.* Results.* After 8 wk of supplementation FPG, HbA1c, HOMA-IR, and insulin levels remained unchanged in the probiotic and placebo groups. The comparison between the two groups showed no significant differences with FPG and HbA1c, but there were significant differences in insulin levels and HOMA-IR (16.6 ± 5.9; 3.7 ± 1.5, resp.). Unlike the levels of IFN-g (19.21 ± 16.6), there was a significant decrease in levels of IL-6 (3.81 ± 0.7), TNF-*α* (3.10 ± 1.1), and hs-CRP (4927.4 ± 924.6). No significant increase was observed in IL-10 (3.11 ± 5.7) in the intervention group as compared with the control group.* Conclusions.* In women with GDM, supplementation with probiotics (VSL#3) may help to modulate some inflammatory markers and may have benefits on glycemic control.

## 1. Introduction

Every year up to 3 million US pregnant women are affected by gestational diabetes mellitus (GDM), diabetes mellitus during pregnancy, and it still has an increasing progress in pregnant women society [1]. GDM has unfavorable effects on both mother and the baby which could be categorized into short term and long term morbidities. Preeclampsia and delivery by cesarean section are the short term outcomes of GDM in mothers while hypoglycemia, excessive adiposity, shoulder dystocia, and macrosomia (baby's birth weight > 8 pounds 13 ounces (4000 g)) are life threatening short term effects of GDM on newborns [2–5]. In long term, both mother and the baby might face with difficulties including increased risk of obesity, cardiovascular diseases, and metabolic disorders such as type 2 diabetes mellitus [4]. There are many papers which mention a 20% higher risk of type 2 diabetes mellitus within nine years after delivery so the women with GDM needs more intervention for reducing future risk of type 2 diabetes mellitus [6]. On the other hand, alteration in insulin resistance in GDM predisposes women to inflammation that causes increased level of inflammatory markers like high sensitive C-reactive protein (hs-CRP) and fibrinogen [7].

Recently the role of the gut microbiota as a modulator of metabolic and inflammatory processes has been investigated. The gut microbiota is affected by many factors like antibiotic therapy [8], dietary patterns [9], weight loss [10], and pregnancy [4]. Intake of probiotics is a safe alternative for normalizing the gut microbiota. In 2002, the Joint FAO (Food and Agricultural Organization of the United Nations)/WHO (World Health Organization) Expert Consultation was held and the latest definition for probiotics was reported as “live microorganisms which, when administered in adequate amounts, confer health benefits on the host” [11]. The most important factors in efficacy of probiotics treatment in inflammatory and metabolic processes are genus and strains supplied in the probiotics. For example, some strains of lactic acid bacteria (LAB) could modulate inflammatory and hypersensitivity process [12]. VSL#3 which was used as an intervention in this trial contained eight strains of lactic acid bacteria (*Streptococcus thermophilus*,* Bifidobacterium breve*,* Bifidobacterium longum*,* Bifidobacterium infantis*,* Lactobacillus acidophilus*,* Lactobacillus plantarum*,* Lactobacillus paracasei*, and* Lactobacillus delbrueckii* subsp. Bulgaricus). This is the first time to investigate the effect of the specific probiotic on glycemic control and inflammatory status among the GDM subjects, although it has been previously used in other population groups [13–17]. So far the exact mechanisms of probiotics in alteration of the gut microbiota are not known [18].

In 2013, a prospective, double-blind randomized controlled trial was conducted on pregnant women which were recruited during 2 years. They received probiotics or a placebo capsule from 16 weeks of gestation until delivery. The results showed more than 50% change in gestational diabetes markers in pregnant women [4].

As previously mentioned, the VSL#3 probiotic has been used in other population groups to influence various blood parameters [13–17] but has not yet been studied in a GDM population. Therefore, a clinical trial was conducted in order to find the effects of this probiotics mixture on inflammatory markers and glycemic control in pregnant women with GDM.

## 2. Materials and Methods

On the basis of the sample size formula suggested for the similar randomized clinical trials [19] and considering types I and II error of 5% (*α* = 0.05) and 20% (*β* = 0.2; Power = 80%), respectively, and serum insulin level as a key variable, we reached the sample size of 44 women for each group. Of the 96 participants who were referred to Tuba endocrine clinic (Sari, Iran) and assessed for study eligibility, 89 participants met inclusion criteria. All 89 consecutive patients diagnosed with gestational diabetes in our department from May 2014 until October 2015 were asked to participate in our study. Among them, 82 agreed to participate and were randomly assigned by blocked randomization, at a ratio of 1 : 1, to take VSL#3 probiotic capsule (*n* = 41) or placebo (*n* = 41). Random assignment was done by using the random numbers generated by computer and a trained personnel at the endocrine clinic performed randomization. VSL#3 is a freeze-dried pharmaceutical probiotic preparation containing 112.5 × 109 CFU/capsule of eight strains of lactic acid bacteria (*Streptococcus thermophilus*,* Bifidobacterium breve*,* Bifidobacterium longum*,* Bifidobacterium infantis*,* Lactobacillus acidophilus*,* Lactobacillus plantarum*,* Lactobacillus paracasei*, and* Lactobacillus delbrueckii* subsp. Bulgaricus), microcrystalline cellulose, stearic acid, magnesium stearate, and vegetable capsule (hydroxypropyl methylcellulose), silicon dioxide. Some dairy ingredients are used in the culture medium but are removed during manufacturing. Identical-looking placebo capsules containing 40 mg microcrystalline cellulose were used for blinding. The capsules were stored at 2 to 8°C prior to distribution and the subjects were instructed to refrigerate the capsules.

GDM diagnosis will be based on the following criteria: 2 h, 75 g OGTT: fasting venous plasma glucose level, 5.5 mmol·L^−1^ or higher or 75-g oral glucose tolerance test [OGTT] 2-hour venous plasma glucose level, 8.0 mmol·L^−1^ or higher [20]. Exclusion criteria were an unwillingness to follow a prescribed diet, pre-GDM (either type 1 or type 2 DM), the need for insulin treatment, and pregnancy comorbidities other than obesity, hypertension, and/or dyslipidemia.

The study was approved by and performed under the guidelines of the Research Ethics Committee of Mazandaran University of Medical Sciences, Iran, and a written consent was obtained from all subjects.

The allocation of the intervention or control group was concealed from the researchers and the probiotic and placebo boxes had an identical appearance. Neither the subjects nor the investigators were aware of the treatment assignments in this double-blinded study. Ten days before the beginning of the trial, all patients refrained from eating yogurt or any other fermented foods or supplements. Over 8 wk, the intervention and placebo groups consumed probiotic and placebo capsules twice a day, respectively. All patients were asked, throughout the 8-week trial, to maintain their usual dietary habits and lifestyle and to avoid consuming any bacteria supplement other than that provided to them by the researchers and any fermented foods.

Arrangements were made so that the patients would receive a 2-week supply of their probiotic or placebo capsules every two weeks. Compliance with the consumption guidelines was monitored by telephone interviews once a week. Information on food consumption, anthropometric measurements, and fasting blood samples were collected at the beginning and at the end of the trial. Nutrient intakes during 3 d were estimated using a 24-hour dietary recall at the beginning and at the end of the study. Three-day averages of macronutrient and micronutrient intakes were analyzed by Nutritionist 4 software (First Databank, Hearst Corp, San Bruno, CA, USA).

Anthropometric measurements were recorded by trained personnel. Body weights were measured using a scale (Seca, Hamburg, Germany) with 0.1 kg accuracy without shoes and with minimum clothing. Heights were measured using a stadiometer (Seca) with 0.1 cm accuracy without shoes. BMI was calculated by dividing body weight (kilograms) by height (meters) squared. Blood samples were collected from the antecubital vein while fasting (12-hour overnight fast) to determine baseline glucose and insulin and then repeated after 8 weeks of treatment. The serum samples were separated from whole blood by centrifugation at 3500 rpm for 10 min (Avanti J-25, Beckman, Brea, CA, USA). The serum and whole blood samples were frozen immediately at −70°C until the assay. Fasting blood glucose was measured using the standard enzymatic method with a Parsazmun kit (Karaj, Iran). Glycated hemoglobin (HbA1c) was measured in the whole blood by cation exchange chromatography with a NycoCard HbA1c kit (Oslo, Norway). Insulin concentration was determined by a chemiluminescent immunoassay using a Liaison analyzer (DiaSorin, Saluggia, Italy). Insulin resistance was assessed using the homeostatic model assessment of insulin resistance (HOMA-IR) formula. Inflammatory parameters containing high-sensitivity C-reactive protein (hs-CRP) and interleukin-6 (IL-6) were determined by the ELISA technique using commercially available kits (Quantikine Human, R&D Systems, Minneapolis, MN) and Interferon gamma  (IFN-*γ*), tumor necrosis factor-*α* (TNF-*α*), and interleukin-10 (IL-10) levels were measured with an enzyme-linked immunosorbent assay kit (eBioscience, San Diego, California) in accordance with the manufactures' instructions.

The present study was conducted according to the guidelines laid down in the declaration of Helsinki and the protocol has been approved by the local ethics committee. The steering committee was established to monitor the trial.

SPSS version 17.0 (SPSS Inc., Chicago, IL, USA) computer software was used for the statistical analysis. The variables were expressed as means SD. After testing data for normality, Student's *t*-test and paired Student's *t*-test were used to compare values between baseline and the end of the study. *P* < 0.05 was considered significant for all the data analyzed.

## 3. Results

There were two women in the study group and five in the control group who required insulin treatment because glycaemic goals [21] were not reached with diet alone; these individuals were excluded from the study. Furthermore, two woman in the study group and one women in the control group were excluded because they delivered prematurely before 35 weeks of gestation. Participants did not report any adverse effects or symptoms.

**Figure 1:**
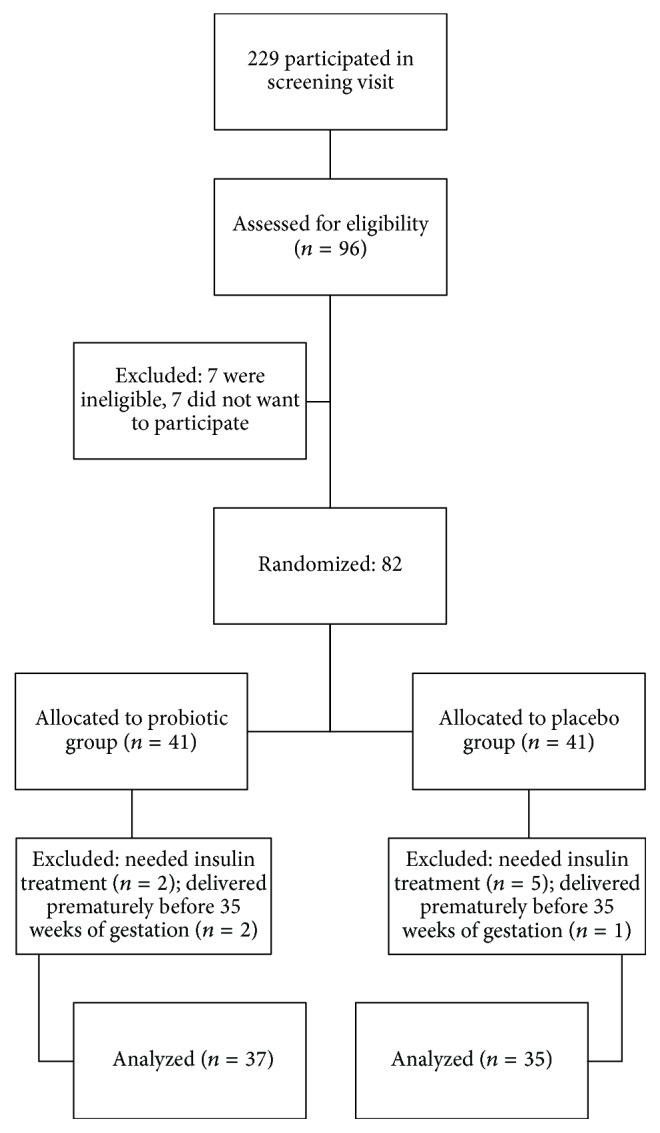
Flow diagram of the study.

The final data analyses included 37 women treated with probiotic capsule and 35 treated with placebo (Figure 1). The baseline characteristics (age, height, BMI, prepregnancy weight, weight at baseline and end of trial, and oral glucose tolerance test values containing 1 h-2 h and 3 h fasting glucose) of the 72 evaluable patients in the two groups were similar and there were no significant differences in the intention to treat baseline characteristics between the two groups, as shown in Table 1. The dietary intake of macronutrients among participants which is shown in Table 2 indicated that there were no significant differences in energy or macronutrient intake. In the other hands, Participants dietary composition did not significantly change during the study period (*P* > 0.05).

With intention to treat analysis and after 8 wk of supplementation FPG, HbA1c, HOMA-IR, and insulin levels remained unchanged in the probiotic and placebo group (*P* > 0.05). The comparison between the two groups showed that there were no significant differences with some glycemic parameters containing FPG and HbA1c (*P* > 0.05), but there were significant differences in insulin levels and HOMA-IR which were calculated based on the power of 80% and *α* = 5% (16.6 ± 5.9; 3.7 ± 1.5, resp.) (Table 3).

There was a significant decrease in levels of IL-6 (3.81 ± 0.7), TNF-*α* (3.10 ± 1.1), and hs-CRP (4927.4 ± 924.6). Reductions in levels of IFN-g (19.21 ± 16.6) were not significant.

No significant increase was observed in IL-10 (3.11 ± 5.7) in the intervention group as compared with the control group after 8 wk (*P* > 0.05) (Table 3).

## 4. Discussion

There are some data from studies, mostly animal studies, which suggest mechanisms whereby probiotics may improve glycemic control, insulin resistance, and inflammatory status [22, 23].

To the best of our knowledge, no study has evaluated the impact of VSL#3 probiotics on inflammatory and glycemic status in patients with GDM. The present study suggests that supplementation with VSL#3 with different subspecies of* Lactobacillus*,* Bifidobacterium*, and* Streptococcus* may have a slight favorable effect on glycemic status. In the study, using the supplement product could not significantly affect FPG and HbA1c but prevented the rise in serum insulin concentration and increase in insulin resistance.

Several studies on the effects of different strains of probiotics on glycemic status in patients with GDM have yielded inconsistent results. One study in Finland was conducted to assess the efficacy of probiotic supplement in reducing the risk of GDM in pregnant women. In the study, 256 women were randomized in the first trimester of pregnancy to receive probiotic supplement (*Lactobacillus rhamnosus* ATCC 53103 and* Bifidobacterium lactis* Bb-12), while the control group received placebo. It has been concluded that the probiotic intervention has a positive effect of glycemic status because blood glucose concentrations were the lowest in the women receiving the probiotic intervention [24]. Our results with regard to prevention of increased serum insulin concentration and development of insulin resistance are similar to those of Asemi and colleagues [19], who showed that consumption of probiotic yogurt which was fermented with* Streptococcus thermophilus* and* Lactobacillus bulgaricus* and subsequently supplemented with* Bifidobacterium lactis* Bb-12 and* Lactobacillus acidophilus* LA5, for 9 weeks in the third trimester of pregnancy, prevented the increase in serum insulin levels and the development of insulin resistance. They showed that the probiotic yogurt could not significantly affect FPG, systolic and diastolic blood pressures compared with the conventional yogurt.

In another study, the rate of GDM was significantly reduced to 13% in women receiving specific probiotics. The results during the last trimester of pregnancy confirmed that blood glucose concentrations were statistically significantly the lowest in the probiotics group; better glycemic status in intervention group was confirmed by the low insulin concentration and homeostasis model assessment, in addition to the reduced risk of elevated glucose concentration compared with the placebo group. The researchers implied that benefits achieved by probiotic intervention were maybe the result of direct effect of modulation of host metabolism by probiotics and indirect effect of modified gut microbiota composition [25].

Lindsay et al. in a double-blind, placebo-controlled trial assessed the impact of a probiotic intervention in pregnant women with an early pregnancy body mass index from 30.0 to 39.9. The primary outcome was the change in fasting glucose between groups from preintervention to postintervention. Secondary outcomes were the incidence of gestational diabetes and neonatal anthropometric measures. No differences in primary and secondary outcome were detected between the study groups, which is in agreement with our results in regard to the primary outcome [26].

In a recent study of 149 women with GDM, maternal fasting glucose decreased from preintervention to postintervention within both groups, but no difference in baseline-adjusted postintervention levels between intervention group with probiotic supplement subspecies* Lactobacillus salivarius* UCC118 and placebo group was detected [27]. The researchers described that the probiotic did not appear to have any beneficial glycemic effect or any improvement of pregnancy outcomes.

One of the common points of these studies was to describe exact mechanism involved in the protective effects of probiotics which is unclear. Inconsistent results of the effect of probiotics on glycemic status described above are maybe because of the strain-specificity of these microorganisms. Every strain of probiotic has its own specific and unique local and systemic immunomodulatory effects which is because of the specific conditions associated with altered gut barrier function [28, 29].

Another outcome of VSL#3 supplementation in patients with GDM was an improvement in inflammatory status. As the inflammatory signalling pathways involved are causally linked to insulin resistance [30], it is speculated that improvement of inflammatory status could be contributed to diabetes control [31]. Therefore, assessing the inflammatory status of patients may show the diabetes status indirectly. The results of the study provide insight into the anti-inflammatory effects of probiotic supplements in patients with gestational diabetes. Our results have shown that probiotic supplements significantly decreased IL-6, TNF-*α*, and hs-CRP in the intervention group as compared with the control group. The reductions in IFN-g and the increase in IL-10 in the intervention group were not significant.

The results were in close agreement with the results as assessed by Matsuzaki et al. showing that* Lactobacillus casei* significantly decreased plasma levels of glucose and proinflammatory cytokines (such as IL-2 and IFN-*γ*) in none-insulin-dependent diabetic mice after 16 weeks [32].

The results are in line with some other studies; Mazloom et al. showed that interleukin-6 (IL-6) was reduced while CRP levels were elevated but the change was not statistically significant. Twetman et al. showed that chewing gums (with two strains:* Lactobacillus reuteri* ATCC 55730 and ATCC PTA 5289) significantly reduced TNF-*α* level [33], although this did not have any significant effect on IL-6 level in healthy adults [34]. Mohamadshahi et al. also reported a significant decrease of serum levels of TNF-*α* by using a probiotic yogurt enriched with* Bifidobacterium animalis* subsp. Lactis Bb12 (DSM 10140) and* Lactobacillus acidophilus* strain La5 [35]. Asemi et al. in two separate studies with two different interventions (probiotic and synbiotic) in patients with type 2 diabetes reported that consumption of the probiotic yogurt appeared to attenuate low-grade inflammation by a significant decrease in sensitive CRP levels in pregnant women [19, 36]. All of the above studies have suggested that using of different species of probiotics may lead to inhibition of production of various proinflammatory cytokines.

However, there are some studies with inconsistent results in contrast with our study. Hatakka et al. considered a nonsignificant change of IL-6 and TNF-*α* level in rheumatoid arthritis patients who take capsules of* Lactobacillus rhamnosus* LC70536. They showed that serum IL-1 beta increased slightly in the LGG group, but no differences were seen in IL-6, TNF-alpha, MPO, IL-10, or 1L-12 [37]. Asemi et al. showed that the intake of probiotic yogurt for 9 weeks brought about a decrease in the serum hs-CRP level but did not decrease TNF-*α* concentration [38].

The mechanism by which probiotic intake results in altered inflammatory and glycemic status is the gut microbiota. There are several studies which examined gut microbiota composition in diabetes or obesity on microbial flora [39–42]. Recent studies have shown that the association between gut bacteria can influence inflammatory state of patients through the component of the Gram-negative bacterial cell walls, which is called popolysaccharide (LPS). This factor can trigger the inflammatory process by binding to the CD14 toll-like receptor-4 (TLR-4) complex. It is confirmed that deletion of TLR-4 can prevent the high-fat diet-induced insulin resistance [43].

Lee et al. considered a decline in TNF-*α* concentration by* Lactobacillus* HY and they declared that it may be because of the inhibition of trinitrobenzene sulfonic acid and consequently inhibition of TNF-*α* gene expression [44]. Another mechanism for anti-inflammatory effect of probiotics is through NF-K*β* signalling pathway. In a study,* Bifidobacterium longum* decreased TNF-*α* concentration by suppressing NF-K*β* activation of lamina propria mononuclear cells which will lead to downregulation of TNF-*α* production [45].

Therefore, the mechanism of probiotics in GDM could be summarized as altering the properties and profile of gut microbiota to a balanced situation which will lead to an improvement of intestinal permeability function and to a regulated concentration of proinflammatory mediators. However, because of the diversity of human gut microbiota and because of the strain-specificity and multifunctionality of various species of probiotic, it is essential to clarify the complex mechanisms of action that contribute to the effects of probiotics intakes on secretion of proinflammatory mediators, which are only lately being unraveled.

Our study had some limitations. The duration of the intervention was 8 weeks and we were unable to administer the supplement for a longer period due to budget limitations. We were unable to measure fasting glucose and serum insulin levels repeatedly. We could not assess the pregnancy and neonatal outcome data. We did not assay other inflammatory or anti-inflammatory biomarkers. The small sample size for detecting the difference in the primary outcomes is another limitation. We also did not evaluate microbial composition of gut and feces.

## 5. Conclusions

According to the results of this trial, clear differences in glycemic status and especially inflammatory mediators are revealed in this study. But we cannot draw any firm conclusion about using probiotics in health improvement profile of GDM patients, clinically. Future studies and further randomized trials of probiotics on pregnancy outcomes are suggested to fully elucidate the potential effects of probiotics in GDM patients.

## Figures and Tables

**Table 1:** Baseline characteristics of the intention-to-treat population.

Variables	Probiotic group (*n* = 41)	Control group (*n* = 41)	*P* value
Age (year)	32.4 ± 3.1	31.9 ± 4.0	0.20
Height (cm)	162.1 ± 4.8	160.9 ± 4.6	0.27
BMI (kg/m^2^)	26.8 ± 2.7	27.4 ± 3.1	0.19
Oral glucose tolerance test values (mmol/L)			
Fasting glucose	5.2 ± 0.6	4.9 ± 0.7	0.34
1-hour glucose	11.8 ± 1.3	11.1 ± 1.6	0.33
2-hour glucose	10.4 ± 1.2	10.2 ± 1.4	0.39
3-hour glucose	7.8 ± 1.8	7.6 ± 1.6	0.46
Prepregnancy weight (kg)	70.4 ± 7.3	69.8 ± 9.2	0.37
Weight at study baseline (kg)	72.9 ± 6.6	73.3 ± 8.6	0.47
Parity ≥ 1 (N)	16	15	0.64
Gestational age (weeks)	26.4	26.6	0.49
Weight at end of trial (kg)	74.9 ± 7.7	75.3 ± 8.3	0.34

BMI, body mass index; data are the means ± SD; obtained from an independent *t*-test.

**Table 2:** Dietary intakes of the intention-to-treat participants at baseline and at the end of the study.

Variables	Period	Probiotic group (*n* = 41)	Control group (*n* = 41)	*P* value^*∗*^
Energy (kcal/d)	Initial	2331 ± 132	2382 ± 149	0.22
	End	2288 ± 117	2310 ± 169	0.13

Carbohydrate (g/d)	Initial	310.8 ± 38.8	318.1 ± 39.1	0.23
	End	302.4 ± 38.2	309.4 ± 39.8	0.29

Protein (g/d)	Initial	79.3 ± 11.4	80.4 ± 13.4	0.22
	End	76.2 ± 14.2	78.7 ± 13.9	0.19

Total fat (g/d)	Initial	77.2 ± 12.6	79.6 ± 8.1	0.34
	End	73.3 ± 13.2	74.1 ± 10.8	0.41

Dietary fiber (g/d)	Initial	13.8 ± 3.2	14.6 ± 3.8	0.25
	End	13.1 ± 4.4	13.6 ± 3.9	0.29

PUFA (g/d)	Initial	20.9 ± 4.5	21.8 ± 3.9	0.36
	End	25.1 ± 5.7	23.7 ± 6.1	0.19

MUFA (g/d)	Initial	21.9 ± 6.2	23.8 ± 5.8	0.48
	End	19.8 ± 8.8	20.7 ± 6.2	0.56

Zinc (mg/d)	Initial	11.8 ± 3.1	9.8 ± 2.1	0.32
	End	10.8 ± 2.6	10.4 ± 3.2	0.47

Copper (mg/d)	Initial	1.39 ± 0.3	1.42 ± 0.5	0.29
	End	1.49 ± 0.5	1.55 ± 0.7	0.31

Data are presented as mean (SD); SFA: saturated fatty acid; PUFA: polyunsaturated fatty acid; MUFA: monounsaturated fatty acid; *∗*: obtained from Student's *t*-test.

**Table 3:** Characteristics of patients who received probiotic or placebo, at baseline and after 8 weeks from diagnostic oral glucose tolerance test (OGTT).

	Probiotic group (*n* = 37)			Control group (*n* = 35)			*P* value
	First week	Eighth week	Change	First week	Eighth week	Change	
Weight in pregnancy (kg)	73.2 ± 7.8	75.7 ± 8.3	2.5 ± 0.3	73.5 ± 9.6	76.2 ± 9.8	2.7 ± 0.4	0.21
FPG (mg/dL)	91.6 ± 4.3	89.3 ± 3.4	−2.3 ± 4.1	93.7 ± 3.1	88.9 ± 4.4	−4.8 ± 3.6	0.42
Insulin (*μ*IU/mL)	19.1 ± 4.2	16.6 ± 5.9^*∗*^	−2.5 ± 5.1	18.7 ± 5.8	22.3 ± 4.9	3.6 ± 5.5	0.04
HOMA-IR	4.3 ± 1.2	3.7 ± 1.5^*∗*^	−0.6 ± 1.4	4.4 ± 1.3	4.9 ± 1.2	0.5 ± 1.2	0.03
HbA1c (%)	4.8 ± 0.6	4.7 ± 0.5	−0.1 ± 0.2	4.60 ± 0.1	4.68 ± 0.3	0.08 ± 0.2	0.44
hs-CRP (ng/mL)	5723.4 ± 1832.0	4927.4 ± 924.6^*∗*^	−796.0 ± 1087.2	5021.2 ± 1301.1	5996.5 ± 1118.8	975.3 ± 1121.2	0.03
IL-10 (pg/mL)	2.37 ± 4.6	3.11 ± 5.7	0.74 ± 4.4	3.78 ± 6.3	3.38 ± 5.8	−0.4 ± 5.1	0.54
IFN-c	16.84 ± 14.9	19.21 ± 16.6	2.37 ± 14.2	18.34 ± 13.4	21.11 ± 15.8	2.77 ± 11.8	0.67
IL-6 (pg/mL)	4.25 ± 0.9	3.81 ± 0.7^*∗*^	−0.44 ± 0.5	4.38 ± 0.64	4.71 ± 0.53	0.33 ± 0.42	0.04
TNF-*α* (pg/mL)	3.72 ± 1.0	3.10 ± 1.1^*∗*^	−0.62 ± 1.0	3.62 ± 1.2	4.07 ± 0.9	0.45 ± 0.8	0.04

All continuous variables are expressed as mean ± SD.

^*∗*^Statistical significance (*P* < 0.05), obtained from independent samples *t*-test, indicates between group differences. HOMA-IR, homeostasis model assessment of insulin resistance. FPG, fasting plasma glucose. HbA1c, glycated Hb.
